# Preprocedural vascular sheath insertion reduces hospital mortality in high risk PCI patients

**DOI:** 10.1038/s41598-026-36613-z

**Published:** 2026-02-13

**Authors:** Bin Sun, Chuang Liu, Meiyan Zhou, Ning Zhen, Ming Liu, Zhen Peng, Yueyue Liu, Yan Zhang, Qian Liu, Guowei Fu, Liwei Wang

**Affiliations:** 1 Department of Anesthesiology, Xuzhou Clinical School of Xuzhou Medical University, Xuzhou Central Hospital, Xuzhou, Jiangsu China; 2 Department of Extracorporeal Life Support Center, Department of Cardiac Surgery, The First Affiliated Hospital of Zhengzhou University, Zhengzhou, Henan China; 3 Operating Room, Xuzhou Clinical School of Xuzhou Medical University, Xuzhou Central Hospital, Xuzhou, Jiangsu China; 4 Department of Cardiology, Xuzhou Clinical School of Xuzhou Medical University, Xuzhou Central Hospital, Xuzhou, Jiangsu China; 5 Department of Ultrasonography, Xuzhou Clinical School of Xuzhou Medical University, Xuzhou Central Hospital, Xuzhou, Jiangsu China; 6 Department of Radiology, Xuzhou Clinical School of Xuzhou Medical University, Xuzhou Central Hospital, Xuzhou, Jiangsu China

**Keywords:** Percutaneous coronary intervention, Vascular sheath, Mechanical circulatory support, High-risk PCI, Mortality, Neurological outcomes, Cardiac device therapy, Interventional cardiology

## Abstract

High-risk patients undergoing percutaneous coronary intervention (PCI) are prone to serious complications, such as ischemia-reperfusion injury, hemodynamic collapse, and cardiogenic shock. While mechanical circulatory support (MCS) may mitigate these risks, its prophylactic use is limited by increased healthcare costs and device-related complications. Preemptive insertion of femoral arteriovenous sheaths, a novel strategy designed to facilitate rapid MCS deployment in emergency situations, may address these concerns, but its clinical benefits remain unclear. In this multicenter retrospective study, we analyzed outcomes from 443 high-risk PCI patients to evaluate whether pre-inserting vascular sheaths before PCI improves patient outcomes. After propensity score matching, 110 matched pairs of patients were compared. Pre-insertion of vascular sheaths was associated with lower all-cause in-hospital mortality (4.5% vs. 18.2%; OR, 0.21; 95% CI, 0.08 ~ 0.59; *P* = 0.003) and a lower rate of poor neurological outcomes (9.1% vs. 19.1%; OR, 0.42; 95% CI, 0.19 ~ 0.96; *P* = 0.037). No significant differences were observed regarding the incidence of cardiogenic shock, MCS usage, or vascular complications. Our findings indicate that routine pre-insertion of vascular sheaths prior to high-risk PCI was associated with improved survival and better neurological outcomes, without a detectable increase in procedural complications, supporting its broader implementation in clinical practice.

**Supplementary Information:**

The online version contains supplementary material available at 10.1038/s41598-026-36613-z.

## Introduction

Coronary artery disease (CAD) continues to be the leading cause of heart disease worldwide, contributing significantly to both morbidity and mortality despite advances in medical and interventional therapies^1,2^. Percutaneous coronary intervention (PCI) has emerged as a highly effective treatment modality for CAD^3^, offering a means to restore blood flow to ischemic regions of the heart^4^. However, the PCI procedure can trigger severe complications, including ischemia-reperfusion injury, bleeding, and hemodynamic instability, particularly in high-risk patients^5^. These complications heighten the risk of cardiovascular collapse, cardiogenic shock, and, in severe cases, death^6,7^, underscoring the critical need for improved management strategies in this vulnerable group.

For high-risk PCI patients, the integration of mechanical circulatory support (MCS) devices such as extracorporeal membrane oxygenation (ECMO) and intra-aortic balloon pumps (IABP) plays a pivotal role in stabilizing hemodynamics during the procedure^8^. These devices enhance coronary perfusion and systemic circulation, particularly in cases of acute decompensation^9,10^. However, the routine use of MCS has been met with skepticism^9,11–13^, largely due to concerns regarding device-related complications, the invasiveness of the intervention, and the increased costs associated with MCS deployment^14,15^. Despite these challenges, MCS remains crucial for managing high-risk PCI patients, and novel strategies are being explored to facilitate its timely initiation.

In response to these concerns, pre-insertion of femoral arteriovenous sheaths before PCI has been proposed as a novel strategy to expedite the initiation of MCS. This technique involves preemptively establishing vascular access by placing sheaths, thereby enabling immediate deployment of MCS devices in emergency situations and potentially improving outcomes in high-risk PCI patients. However, the clinical benefits of this approach remain understudied. Therefore, the primary objective of this study is to determine whether pre-inserting vascular sheaths before PCI reduces all-cause in-hospital mortality in high-risk patients. Additionally, secondary outcomes include MCS device utilization, incidence of cardiogenic shock, and occurrence of poor neurological outcomes.

## Methods

### Study population

This multi-center retrospective cohort study included all complex high-risk indicated patients who underwent PCI between January 2018 and December 2022 at Xuzhou Central Hospital (Jiangsu, China) and the First Affiliated Hospital of Zhengzhou University (Henan, China). High-risk patients were defined as those with a pre-procedural ejection fraction below 35% and/or requiring atherectomy, treatment of an unprotected left main artery, or treatment of multiple vessels (≥ 2 vessels)^16^. Inclusion required an ejection fraction below 35% combined with at least one additional high-risk criterion. Patients were excluded if they lacked essential clinical records, underwent PCI via femoral artery, received MCS prior to PCI, experienced cardiogenic shock, cardiac arrest, or other severe events before PCI, or had a pre-procedural cerebral performance category (CPC) score of ≥ 3.

The study was registered with the China Clinical Trial Registry (ChiCTR2300076626) and approved by the ethics committees of both participating institutions: the ethics committee of Xuzhou Central Hospital (Approval No. XZXY-LK-20231010-0159) and the ethics committee of the First Affiliated Hospital of Zhengzhou University (Approval No. 2023-KY-0638-002). Due to the retrospective nature of the study, the ethics committee of Xuzhou Central Hospital and the ethics committee of the First Affiliated Hospital of Zhengzhou University waived the need of obtaining informed consent. All procedures were performed in accordance with the Helsinki Declaration and the policies of the Nature Portfolio journals.

### Pre-insertion techniques

The exposure measure in this study was the insertion of vascular sheaths prior to PCI, without any specific restrictions. Upon entering the catheterization room, patients scheduled for sheaths insertion underwent skin preparation around the perineum, followed by disinfection and draping of the groin area. Using ultrasound guidance, the femoral artery was typically punctured first. After successful puncture, a guidewire was inserted to facilitate sheath placement. Once the sheath was positioned, both the sheath core and guidewire were removed, and the catheter was flushed with heparinized saline (25 U/mL). Following a similar procedure, a sheath was also placed in the femoral vein and secured with a sterile adhesive film. Pre-insertion sheaths (Terumo Corporation, Japan) were generally 5–6 F in size (Figure S1).

ECMO and IABP devices were prepared next to the patient for rapid deployment if necessary, as Impella devices were unavailable at our research institution. If a patient developed cardiogenic shock or cardiac arrest during PCI, in addition to standard resuscitation measures, those in the sheath group could undergo rapid ECMO cannulation or IABP insertion via the pre-insertion vascular sheaths. By contrast, patients in the control group required additional vascular puncture for emergency MCS deployment, which often necessitated interruptions in chest compressions (CC) during CPR. If the vascular sheaths were not needed during PCI, they were generally removed 48 h post-procedure using compression.

The number of vessels and lesions treated, as well as the use of adjunctive therapies (e.g., atherectomy devices, medications), were left to the physician’s discretion. The timing of MCS insertion and the choice of MCS device were also at the discretion of the operating physician.

### Data extraction

Data were collected retrospectively by trained investigators using a standardized case report form that included demographic and clinical information from high-risk patients who underwent PCI between January 2018 and December 2022. We recorded details such as age, sex, body mass index (BMI), and New York Heart Association (NYHA) classification. We also documented various comorbidities, including hypertension, diabetes, hyperlipidemia, chronic lung disease, atrial fibrillation, chronic heart failure, and peripheral vascular disease. Additionally, we noted information regarding previous myocardial infarction, previous PCI, and previous coronary artery bypass grafting (CABG). Furthermore, we recorded the clinical diagnoses at the time of PCI, preoperative antiplatelet medication use, and the target vessels involved in the procedures.

### Outcome variables

The primary outcome was all-cause in-hospital mortality, defined as death from any cause occurring during hospitalization. The secondary outcomes included the rate of MCS usage, comprising both IABP and ECMO; the incidence of cardiogenic shock during PCI, defined as a systolic blood pressure below 90 mmHg for more than 30 min, the presence of organ failure, or the need for vasopressor or inotropic therapy; the incidence of poor neurological outcomes, defined as a CPC score of 3 or higher at discharge; and the incidence of lower limb cannulation-associated adverse events, including infections, limb ischemia, and vascular complications. To monitor for lower limb ischemia, pedal pulse, temperature, color, and skin sensitivity were regularly assessed during and in the hours following compression. When ischemia was suspected, vascular Doppler examinations were performed to confirm the diagnosis.

### Statistical analysis

As a retrospective analysis, there was no a priori statistical analysis plan. No statistical power calculation or sample size calculation was performed. Sample size determination relied entirely on existing data retrieved from the medical record systems of two hospitals. Patients were classified into two groups: the sheath group (pre-insertion of vascular sheaths) and the control group (no pre-insertion). Missing values for relevant variables were handled using multiple imputation methods^17^.

Data analysis and graph plotting were performed using IBM SPSS Statistics, version 26.0 (IBM Corp., Armonk, NY, USA) and GraphPad Prism, version 9 (GraphPad Software, San Diego, CA, USA), respectively. Continuous variables are presented as means ± standard deviations or medians (*P*_25_, *P*_75_). They were analyzed using either the student’s t-test or the Mann-Whitney U test, depending on their distribution. On the other hand, categorical variables are expressed in percentages and were assessed with chi-squared tests or fisher’s exact test, as appropriate. Logistic regression models were performed to calculate the odds ratio (OR) with 95% CI for dichotomous outcomes. For all analyses, a two-tailed *P* < 0.05 was considered statistically significant.

### Propensity score matching

The propensity score matching (PSM) method strives to create a dataset where the likelihood of pre-inserting sheaths (or not) is evenly balanced. This method seeks to emulate the circumstances of a genuine randomized trial, guaranteeing uniformity in baseline patient attributes. In our research, the matching was constructed based on a 1:1 ratio using the nearest neighbour method with a calliper width of 0.01 without replacement. The balance of variables between the groups before and after matching was assessed using standardised mean difference (SMD), with a value of less than 0.10 indicating balance.

### Subgroup analyses

To evaluate the consistency of our findings, subgroup analyses and interaction tests were performed. The matched cohort was stratified into subgroups based on age (≤ 65 vs. > 65 years), sex (female vs. male), BMI (≤ 30 vs. > 30 kg/m²), NYHA classification (≤ III vs. IV), hypertension (yes vs. no) and hyperlipidemia (yes vs. no).

### Sensitivity analyses

We conducted additional sensitivity analyses using the complete dataset to verify the stability of results identified within the matched cohort. Variables demonstrating statistical significance (*P* < 0.05) in the univariable analyses, namely cigarette smoking, hypertension, diabetes, peripheral vascular disease, involvement of the left main vessel, and chronic total occlusion (CTO), were subsequently included for adjustment in the multivariable regression model (Table S1).

## Results

### Study population

**Fig. 1:**
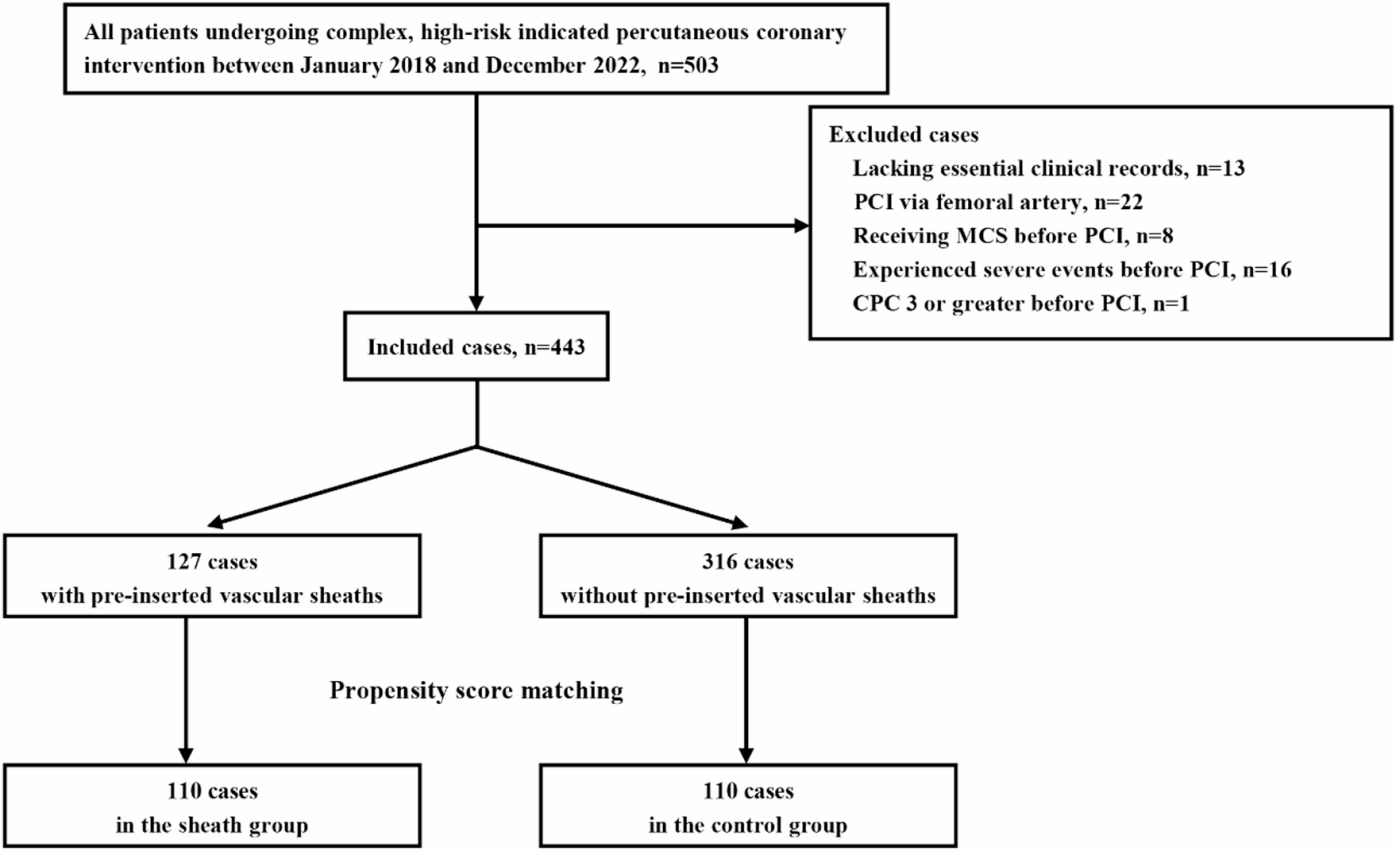
Flow diagram of the case selection process.

The study cohort included 443 high-risk patients who underwent PCI between 2018 and 2022. Of these, 127 patients (28.7%) were in the sheath group, while 316 (71.3%) formed the control group. For further analysis, 110 propensity score-matched pairs were selected. Figure 1 presents the flow diagram of the patients included in this analysis. The study was conducted over five years at two hospitals, which sequentially adopted pre-insertion of vascular sheaths as the primary approach for high-risk PCI beginning in 2020.

### Cohort characteristics

Table 1 and Figure S2 present the baseline demographic and clinical characteristics of high-risk patients before and after PSM. Prior to matching, only 45.8% of the variables (11 out of 24) had an SMD of less than 0.10 between the two groups. The matching process significantly improved variable balance. After matching, the majority of variables achieved an SMD of less than 0.10, except for previous PCI and CABG, confirming that the two groups were well-balanced for subsequent analysis.

**Table 1:** Baseline characteristics before and after propensity score matching.

Variables	Before PSM			After PSM		
	Control group(*n* = 316)	Sheath group(*n* = 127)	SMD	Control group(*n* = 110)	Sheath group(*n* = 110)	SMD
Age, years	62.8 ± 6.4	63.8 ± 7.6	0.139	64.0 ± 6.8	63.3 ± 7.8	0.095
Male	230(72.8)	92(72.4)	0.008	83(75.5)	80(72.7)	0.062
BMI, Kg/m^2	26.9 ± 3.5	27.0 ± 4.0	0.033	26.8 ± 3.5	26.9 ± 3.9	0.043
NYHA classification			0.074			0.049
Ⅱ	62(19.6)	20(15.7)		23(20.9)	19(17.3)	
Ⅲ	127(40.2)	54(42.5)		42(38.2)	46(41.8)	
Ⅳ	127(40.2)	53(41.7)		45(40.9)	45(40.9)	
Cigarette smoking	137(43.4)	39(30.7)	0.263	38(34.5)	37(33.6)	0.019
Hypertension	181(57.3)	86(67.7)	0.216	75(68.2)	71(64.5)	0.077
Diabetes	109(34.5)	30(23.6)	0.241	29(26.4)	30(27.3)	0.020
Hyperlipidemia	235(74.4)	102(80.3)	0.142	87(79.1)	87(79.1)	0.000
Chronic lung disease	8(2.5)	8(6.3)	0.184	4(3.6)	6(5.5)	0.087
Atrial fibrillation	40(12.7)	15(11.8)	0.026	14(12.7)	13(11.8)	0.028
Chronic heart failure	176(55.7)	65(51.2)	0.090	58(52.7)	59(53.6)	0.018
Peripheral vascular disease	13(4.1)	0(0)	0.292	0(0)	0(0)	0.000
Previous myocardial infarction	88(27.8)	24(18.9)	0.212	23(20.9)	23(20.9)	0.000
Previous PCI	31(9.8)	15(11.8)	0.064	16(14.5)	12(10.9)	0.109
Previous CABG	32(10.1)	15(11.8)	0.054	17(15.5)	12(10.9)	0.134
**Clinical diagnosis**			0.011			0.023
STEMI	67(21.2)	27(21.3)		24(21.8)	25(22.7)	
NSTEMI	115(36.1)	45(35.4)		38(34.5)	38(34.5)	
Unstable angina	134(42.4)	55(43.3)		48(43.6)	47(42.7)	
**Preoperative antiplatelet drugs**						
Aspirin	316(100)	127(100)	0.000	110(100)	110(100)	0.000
Clopidogrel	223(70.6)	96(75.6)	0.113	82(74.5)	83(75.5)	0.021
Ticagrelor	93(29.4)	31(24.4)	0.113	28(25.5)	27(24.5)	0.021
**Target vessel**						
Left main	195(61.7)	91(71.7)	0.212	75(68.2)	76(69.1)	0.020
Left anterior descending	280(88.6)	110(86.6)	0.060	95(86.4)	97(88.2)	0.054
Left circumflex artery	213(67.4)	88(69.3)	0.040	77(70.0)	76(69.1)	0.020
Right coronary artery	274(86.7)	102(80.3)	0.172	92(83.6)	90(81.8)	0.048
CTO	85(26.9)	47(37.0)	0.217	33(30.0)	38(34.5)	0.097

BMI, body mass index; NYHA, New York Heart Association; PCI, Percutaneous coronary intervention; CABG, coronary artery bypass grafting; STEMI, ST-segment elevation myocardial infarction; NSTEMI, non-ST-segment elevation myocardial infarction; CTO, chronic total occlusion; PSM, propensity score matching; SMD, standardized mean difference.

### Primary outcome

The all-cause in-hospital mortality rate was significantly lower in the sheath group (4.5%, 5/110) compared to the control group (18.2%, 20/110), with an odds ratio (OR) of 0.21 (95% CI, 0.08 ~ 0.59; *P* = 0.003) (Table 2).

**Table 2:** The study outcomes after propensity score matching.

Outcomes	Control group(*n* = 110)	Sheath group(*n* = 110)	OR (95% CI)	*P*
Primary outcome				
All-cause in-hospital mortality	20 (18.2)	5 (4.5)	0.21 (0.08 ~ 0.59)	0.003
**Secondary outcome**				
Cardiogenic shock (surgery to 48 h postoperatively)	41 (37.3)	38 (34.5)	0.89 (0.51 ~ 1.54)	0.673
MCS usage rate (intraoperative and postoperative)	37 (33.6)	34 (30.9)	0.88 (0.50 ~ 1.55)	0.665
Poor neurological outcomes	21 (19.1)	10 (9.1)	0.42 (0.19 ~ 0.96)	0.037
Lower limb associated adverse events	5 (4.5)	3 (2.7)	0.59 (0.14 ~ 2.53)	0.476
Coronary artery perforation	1 (0.91)	1 (0.91)	1.00 (0.06 ~ 16.19)	1.000

OR: Odds Ratio, CI: Confidence Interval, PCI, percutaneous coronary intervention; MCS, Mechanical circulatory support.

### Secondary outcomes

The incidence of poor neurological outcomes was 9.1% (10/110) in the sheath group compared to 19.1% (21/110) in the control group, with the sheath group showing a significantly lower risk (OR, 0.42; 95% CI, 0.19 ~ 0.96; *P* = 0.037). No significant differences were observed between the groups for cardiogenic shock (34.5% vs. 37.3%; OR, 0.89; 95% CI, 0.51 ~ 1.54; *P* = 0.673), usage of MCS (30.9% vs. 33.6%; OR, 0.88; 95% CI, 0.50 ~ 1.55; *P* = 0.665), or lower limb-associated adverse events (2.7% vs. 4.5%; OR, 0.59; 95% CI, 0.14 ~ 2.53; *P* = 0.476) (Table 2).

### Subgroup analysis

Subgroup analysis of all-cause in-hospital mortality (Table 3) showed significant reductions in mortality for the sheath group across multiple subgroups, including males, females, patients aged ≤ 65 years and > 65 years, BMI ≤ 30 kg/m², on-hours PCI, those with hypertension, and patients classified as NYHA Ⅳ or with hyperlipidemia. Non-significant results were observed for patients with BMI > 30 kg/m², NYHA ≤Ⅲ, off-hours PCI, and those without hyperlipidemia. Importantly, no significant interactions were detected between subgroups, indicating that the benefit of sheath pre-insertion was consistent across diverse patient characteristics. Please note that the subgroup analyses are exploratory. Due to multiple comparisons, there is an increased risk of Type I error. Formal tests for interaction were non-significant, indicating that the treatment effect is likely consistent across subgroups, and overinterpretation of results in any single subgroup should be avoided.

**Table 3:** Subgroup analyses of all-cause in-hospital mortality in the matched cohort.

Variables	*n* (%)	Control group	Sheath group	OR (95% CI)	*P*	*P* for interaction
All patients	220 (100.00)	20/110	5/110	0.21 (0.08 ~ 0.59)	0.003	
Sex						0.401
female	57 (25.91)	7/27	1/30	0.10 (0.01 ~ 0.86)	0.036	
male	163 (74.09)	13/83	4/80	0.28 (0.09 ~ 0.91)	0.034	
Age, years						0.483
≤ 65	121 (55.00)	8/61	1/60	0.11 (0.01 ~ 0.93)	0.042	
> 65	99 (45.00)	12/49	4/50	0.27 (0.08 ~ 0.90)	0.033	
BMI, Kg/m^2^						0.552
≤ 30	179 (81.36)	16/92	3/87	0.17 (0.05 ~ 0.61)	0.006	
> 30	41 (18.64)	4/18	2/23	0.33 (0.05 ~ 2.07)	0.239	
NYHA classification						0.642
≤ Ⅲ	130 (59.09)	10/65	3/65	0.27 (0.07 ~ 1.02)	0.053	
Ⅳ	90 (40.91)	10/45	2/45	0.16 (0.03 ~ 0.79)	0.025	
Hypertension						0.426
No	74 (33.64)	7/35	1/39	0.11 (0.01 ~ 0.90)	0.040	
Yes	146 (66.36)	13/75	4/71	0.28 (0.09 ~ 0.92)	0.036	
Hyperlipidemia						0.735
No	46 (20.91)	6/23	2/23	0.27 (0.05 ~ 1.51)	0.136	
Yes	174 (79.09)	14/87	3/87	0.19 (0.05 ~ 0.67)	0.010	
PCI time						0.990
On-hours PCI	199 (90.45)	19/101	5/98	0.23 (0.08 ~ 0.65)	0.005	
Off-hours PCI	21 (9.55)	1/9	0/12	0.00 (0.00 ~ Inf)	0.997	

OR: Odds Ratio; CI: Confidence Interval; BMI, Body Mass Index; NYHA, New York Heart Association; PCI, percutaneous coronary intervention.

### Sensitivity analysis

In the sensitivity analysis of the full dataset, the all-cause in-hospital mortality rate was 4.7% (6/127) in the sheath group and 17.4% (55/316) in the control group. The sheath group demonstrated significantly lower mortality in both univariable (OR, 0.24; 95% CI, 0.10 ~ 0.56; *P* = 0.001) and multivariable analyses (OR, 0.21; 95% CI, 0.09 ~ 0.51; *P* < 0.001) (Table S2).

## Discussion

Complex high-risk indicated patients undergoing PCI face elevated morbidity, mortality, and procedural failure due to vulnerability to hemodynamic destabilization^18^. Percutaneous MCS can aid selected cases when deployed appropriately^18^. In this observational cohort, pre-insertion of vascular sheaths before high-risk PCI was associated with significantly lower all-cause in-hospital mortality, with a mortality rate of 4.5% in the sheath group compared to 18.2% in the control group, representing a 75% reduction. This benefit was consistent across subgroups stratified by age, gender, and comorbid conditions, and sensitivity analyses confirmed the robustness of these findings. Additionally, pre-insertion of vascular sheaths was associated with fewer poor neurological outcomes (9.1% vs. 19.1%, *P* = 0.037) and showed no significant differences in rates of cardiogenic shock, MCS use, or lower limb complications. Further analysis revealed that IABP cannulation time was 11.0 min shorter in the sheath group compared to the control group (95% CI, −12.5 to −9.5 min, *P* < 0.001), while ECMO cannulation time was 14.7 min shorter (95% CI, −16.7 to −12.7 min, *P* < 0.001). These findings demonstrate that pre-insertion of vascular sheaths was associated with improved procedural efficiency, faster MCS initiation, and lower in-hospital mortality in high-risk PCI, with no significant increase in major complications.

The findings of our study align with and extend prior research that seeks to improve outcomes in high-risk PCI patients. Consistent with Khandelwal et al.^19^, who validated the UK-BCIS CHIP score as a predictive tool for MACCE in high-risk PCI patients, our results emphasize the necessity of innovative approaches tailored for this population. Notably, the pre-insertion of vascular sheaths in our study resonates with the recommendation by the American Heart Association Scientific Statement, which advocates for femoral access strategies in cardiogenic shock settings to enable rapid escalation to temporary MCS if required^14^. The significant reduction in all-cause in-hospital mortality observed in our sheath group may be attributed to the expedited deployment of MCS, such as IABP or ECMO, facilitated by the pre-insertion technique. These findings are in agreement with Atkinson et al.^8^, who highlighted the pivotal role of timely MCS in mitigating complications during high-risk PCI procedures. Furthermore, early and effective vascular access aligns with Nakatsutsumi et al.^20^, who demonstrated that ultrasound-guided cannulation reduces intervention times and associated complications, further underscoring the clinical utility of our approach.

In addition, the reduction in poor neurological outcomes in our study may be attributed to minimized low-flow intervals through timely initiation of MCS, a finding supported by Guy et al.^21^ in the context of extracorporeal resuscitation. The lack of increased vascular complications, such as bleeding, in the sheath group likely reflects advancements in procedural techniques and patient selection, as highlighted by Nguyen et al.^22^. Variability in secondary outcomes compared to prior studies may stem from differences in operator expertise, institutional protocols, or patient characteristics. For example, while Alraies et al.^23^ reported higher bleeding rates among female MCS recipients, our study showed no such gender disparities, likely due to stricter anticoagulation protocols and procedural standardization. These findings underscore the value of innovative strategies, such as pre-inserting vascular sheaths, to enhance outcomes in high-risk PCI patients while ensuring procedural safety.

In our cohort, pre-insertion was associated with shorter time-to-MCS in emergencies, this earlier support may have contributed to improved in-hospital outcomes even though overall MCS use did not differ significantly between groups. Conceptually, pre-insertion adds device and workflow time costs up front but may avert high-cost emergent rescue by enabling faster MCS initiation during decompensation, limiting interruptions to resuscitation, and potentially reducing downstream ICU and neurologic-rehabilitation burden. Additionally, the sheath group experienced negligible interruptions in CC during vascular cannulation, in contrast to the control group, where CC was frequently paused. This aligns with findings by Lauridsen et al., who reported that minimizing CC interruptions during CPR improves survival in cardiac arrest patients^24^. Furthermore, pre-insertion of vascular sheaths allows for seamless insertion of MCS within 48 h post-surgery when needed, adding flexibility to post-procedural care. Operationally, the strategy shifts teams from reactive rescue to proactive readiness, which may enhance efficiency and patient safety during crises. This approach is not only beneficial for complex high-risk PCI patients but may also be valuable for other populations, such as pregnant women with cardiac disease or patients with fulminant myocarditis requiring rapid escalation to MCS. Finally, our use of PSM strengthens the validity of our findings by minimizing bias, offering evidence of the advantages of pre-inserting vascular sheaths in high-risk PCI settings.

## Limitations

Several limitations warrant consideration when interpreting these findings. First, because this was an observational, nonrandomized study, residual confounding remains possible, including confounding by indication. Operators may have selected patients for sheath pre-insertion according to perceived risk, frailty, or technical factors. Although PSM was applied, unmeasured variables such as operator experience, off-hours treatment, and SYNTAX Score II may still bias the observed associations. Second, despite a low overall complication rate, the inherent risks of sheaths pre-insertion, including patient discomfort, infection, bleeding, and vascular injury, remain pertinent. Third, the study was conducted at two tertiary hospitals in China, heterogeneity between centers in vascular-access strategies, anticoagulation protocols, and access to Impella devices may limit generalizability. Fourth, the absence of randomization precludes definitive causal inference. Fifth, our analysis was limited to in-hospital outcomes. Longer-term endpoints, including 30-day and 6-month survival, rehospitalization, and functional status, were not captured and should be evaluated in future prospective studies with registry linkage. Lastly, the relatively small sample size after PSM may have reduced statistical power to detect modest differences in secondary outcomes, including cardiogenic shock and lower-limb complications.

## Conclusion

Pre-inserting vascular sheaths before PCI facilitates rapid initiation of MCS and is associated with improved in-hospital outcomes in high-risk patients. Given its association with lower mortality and better neurological outcomes, this strategy may be a valuable addition to current PCI protocols, particularly for those at the highest risk of complications. However, further research is needed to evaluate long-term outcomes, identify optimal patient selection criteria, and assess the cost-effectiveness of this approach. Such studies will help refine clinical practice and broaden the applicability of this intervention in high-risk PCI patients.

## Supplementary Information

Below is the link to the electronic supplementary material.

Supplementary Material 1

## Data Availability

All data generated or analyzed during this study were included in the published article. Further inquiries about the datasets can be directed to the corresponding author on reasonable request.
