# Supplementary material for: Preprocedural vascular sheath insertion reduces hospital mortality in high risk PCI patients
Source: Sci Rep. 2026 Feb 13;16:8790. doi: 10.1038/s41598-026-36613-z (PMC12982764; doi:10.1038/s41598-026-36613-z)
Supplement: Supplementary file 1 — Supplementary Material 1 [file 41598_2026_36613_MOESM1_ESM.docx]

**Preprocedural vascular sheath insertion reduces hospital mortality in high risk PCI patients**

Bin Sun^1,7^, Chuang Liu^2,7^, Meiyan Zhou^1,7^, Ning Zhen^3^, Ming Liu^4^, Zhen Peng^5^, Yueyue Liu^6^, Yan Zhang^1^, Qian Liu^1^, Guowei Fu^2^
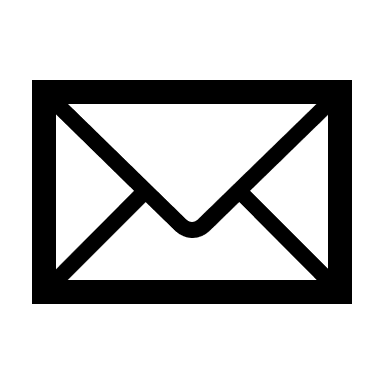
 & Liwei Wang^1^
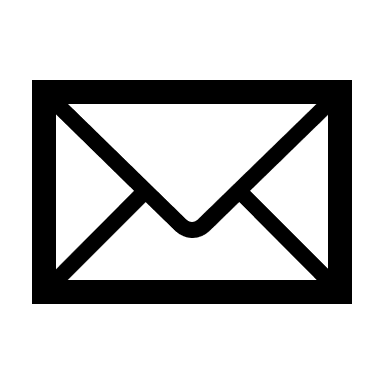


^1^ Department of Anesthesiology, Xuzhou Clinical School of Xuzhou Medical University, Xuzhou Central Hospital, Xuzhou, Jiangsu, China.

^2^ Department of Extracorporeal Life Support Center, Department of Cardiac Surgery, The First Affiliated Hospital of Zhengzhou University, Zhengzhou, Henan, China

^3^ Operating Room, Xuzhou Clinical School of Xuzhou Medical University, Xuzhou Central Hospital, Xuzhou, Jiangsu, China

^4^ Department of Cardiology, Xuzhou Clinical School of Xuzhou Medical University, Xuzhou Central Hospital, Xuzhou, Jiangsu, China

^5^ Department of Ultrasonography, Xuzhou Clinical School of Xuzhou Medical University, Xuzhou Central Hospital, Xuzhou, Jiangsu, China

^6^ Department of Radiology, Xuzhou Clinical School of Xuzhou Medical University, Xuzhou Central Hospital, Xuzhou, Jiangsu, China

^7^ These authors contributed equally: Sun Bin, Liu Chuang and Zhou Meiyan.


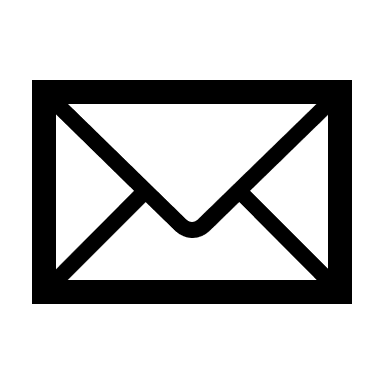
Email: fugwei@163.com; doctorlww@sina.com

Table S1 Baseline characteristics before and after propensity score matching

| **Variables** | **Before PSM** | |  |  | **After PSM** | |  |
| --- | --- | --- | --- | --- | --- | --- | --- |
|  | **Control group（n=316）** | **Sheath group（n=127）** | ***P*** |  | **Control group（n=110）** | **Sheath group（n=110）** | ***P*** |
| Age, years | 62.8±6.4 | 63.8±7.6 | 0.203 |  | 64.0±6.8 | 63.3±7.8 | 0.483 |
| Male | 230(72.8) | 92(72.4) | 0.941 |  | 83(75.5) | 80(72.7) | 0.644 |
| BMI, Kg/m^2 | 26.9±3.5 | 27.0±4.0 | 0.745 |  | 26.8±3.5 | 26.9±3.9 | 0.750 |
| NYHA classification |  |  | 0.635 |  |  |  | 0.755 |
| Ⅱ | 62(19.6) | 20(15.7) |  |  | 23(20.9) | 19(17.3) |  |
| Ⅲ | 127(40.2) | 54(42.5) |  |  | 42(38.2) | 46(41.8) |  |
| Ⅳ | 127(40.2) | 53(41.7) |  |  | 45(40.9) | 45(40.9) |  |
| Cigarette smoking | 137(43.4) | 39(30.7) | 0.014 |  | 38(34.5) | 37(33.6) | 0.887 |
| Hypertension | 181(57.3) | 86(67.7) | 0.042 |  | 75(68.2) | 71(64.5) | 0.568 |
| Diabetes | 109(34.5) | 30(23.6) | 0.026 |  | 29(26.4) | 30(27.3) | 0.879 |
| Hyperlipidemia | 235(74.4) | 102(80.3) | 0.185 |  | 87(79.1) | 87(79.1) | 1.000 |
| Chronic lung disease | 8(2.5) | 8(6.3) | 0.086 |  | 4(3.6) | 6(5.5) | 0.517 |
| Atrial fibrillation | 40(12.7) | 15(11.8) | 0.807 |  | 14(12.7) | 13(11.8) | 0.837 |
| Chronic heart failure | 176(55.7) | 65(51.2) | 0.388 |  | 58(52.7) | 59(53.6) | 0.893 |
| Peripheral vascular disease | 13(4.1) | 0(0) | 0.024 |  | 0(0) | 0(0) | 1.000 |
| Previous myocardial infarction | 88(27.8) | 24(18.9) | 0.050 |  | 23(20.9) | 23(20.9) | 1.000 |
| Previous PCI | 31(9.8) | 15(11.8) | 0.532 |  | 16(14.5) | 12(10.9) | 0.418 |
| Previous CABG | 32(10.1) | 15(11.8) | 0.603 |  | 17(15.5) | 12(10.9) | 0.319 |
| **Clinical diagnosis** |  |  | 0.980 |  |  |  | 0.985 |
| STEMI | 67(21.2) | 27(21.3) |  |  | 24(21.8) | 25(22.7) |  |
| NSTEMI | 115(36.1) | 45(35.4) |  |  | 38(34.5) | 38(34.5) |  |
| Unstable angina | 134(42.4) | 55(43.3) |  |  | 48(43.6) | 47(42.7) |  |
| **Preoperative antiplatelet drugs** |  |  |  |  |  |  |  |
| Aspirin | 316(100) | 127(100) | 1.000 |  | 110(100) | 110(100) | 1.000 |
| Clopidogrel | 223(70.6) | 96(75.6) | 0.287 |  | 82(74.5) | 83(75.5) | 0.876 |
| Ticagrelor | 93(29.4) | 31(24.4) | 0.287 |  | 28(25.5) | 27(24.5) | 0.876 |
| **Target vessel** |  |  |  |  |  |  |  |
| Left main | 195(61.7) | 91(71.7) | 0.048 |  | 75(68.2) | 76(69.1) | 0.884 |
| Left anterior descending | 280(88.6) | 110(86.6) | 0.559 |  | 95(86.4) | 97(88.2) | 0.686 |
| Left circumflex artery | 213(67.4) | 88(69.3) | 0.700 |  | 77(70.0) | 76(69.1) | 0.884 |
| Right coronary artery | 274(86.7) | 102(80.3) | 0.089 |  | 92(83.6) | 90(81.8) | 0.721 |
| CTO | 85(26.9) | 47(37.0) | 0.035 |  | 33(30.0) | 38(34.5) | 0.471 |

BMI, body mass index; NYHA, New York Heart Association; PCI, Percutaneous coronary intervention; CABG, coronary artery bypass grafting; STEMI, ST-segment elevation myocardial infarction; NSTEMI, non-ST-segment elevation myocardial infarction; CTO, chronic total occlusion; PSM, propensity score matching; SMD, standardized mean difference

Table S2 Sensitivity analyses for all-cause in-hospital mortality in the full dataset

| **Outcomes** | **Control group（n=316）** | **Sheath group（n=127）** | **Univariable analysis** | | |  | | **Multivariable analysis** | | |
| --- | --- | --- | --- | --- | --- | --- | --- | --- | --- | --- |
|  |  |  | OR (95% CI) | *P* |  | | OR (95% CI) | | *P* |  |
| All-cause in-hospital mortality | 55 (17.4) | 6 (4.7) | 0.24 (0.10 ~ 0.56) | 0.001 |  | | 0.21 (0.09 ~ 0.51) | | <0.001 |  |

OR: Odds Ratio; CI: Confidence Interval


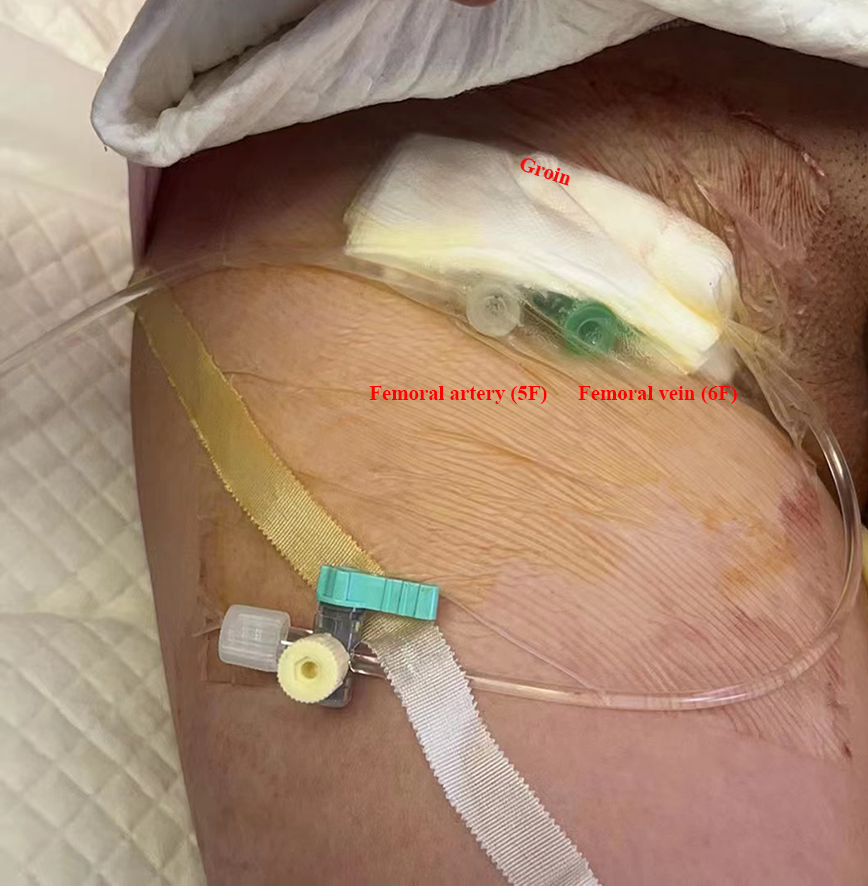


**Figure S1. Pre-insertion of femoral arteriovenous sheaths prior to PCI.** Representative intra-procedural photograph showing preemptive placement of femoral arterial and venous sheaths (5F–6F, Terumo, Japan) under ultrasound guidance prior to PCI. Sheaths were flushed with heparinized saline (25 U/mL) and secured with sterile film to enable rapid initiation of mechanical circulatory support (IABP or VA-ECMO) in the event of hemodynamic decompensation; if not required, they were typically removed within 48 h post-procedure.


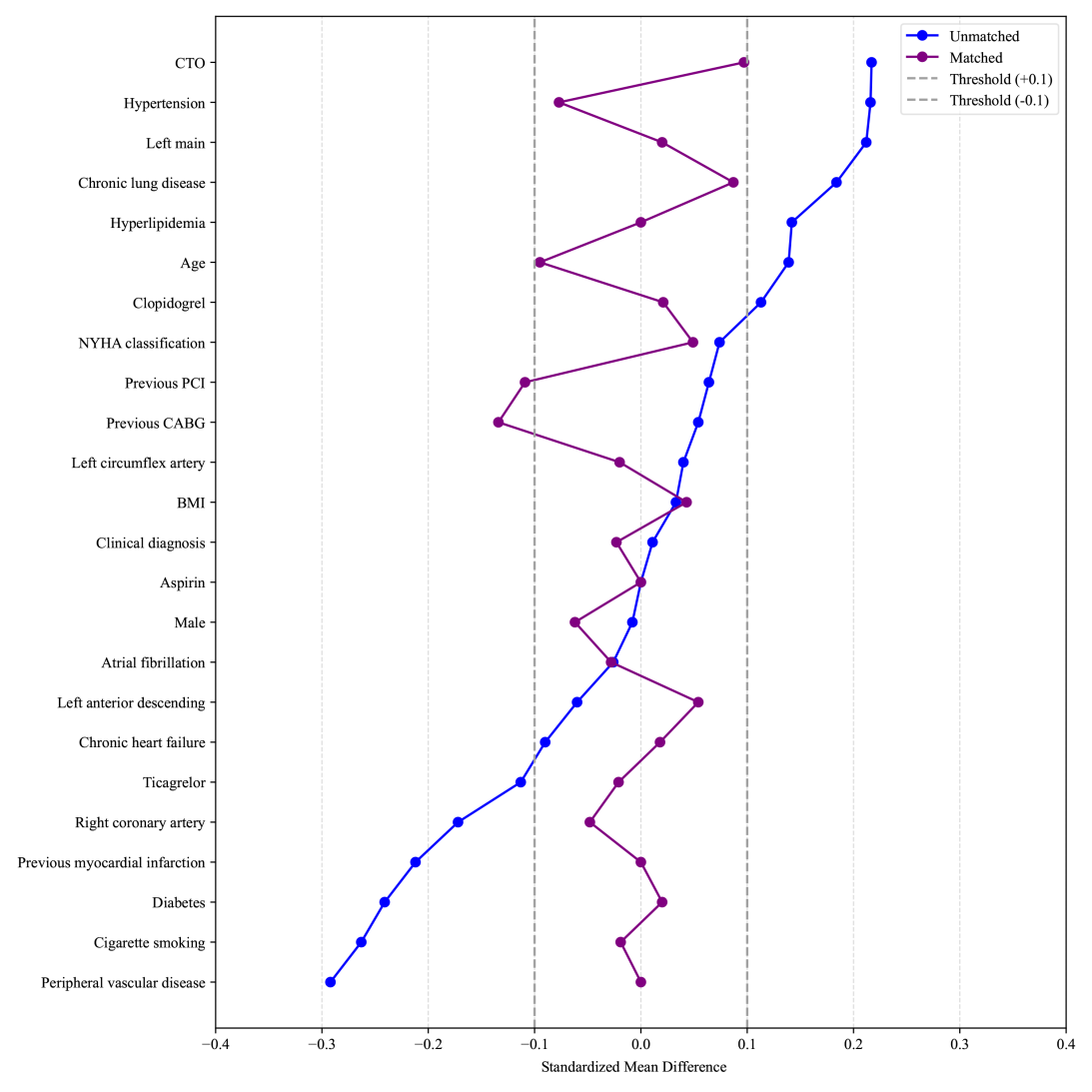


**Figure S2. Standardized mean differences in before and after propensity score matching.** Standardized mean differences (SMD) for baseline characteristics in the unmatched and matched cohorts are displayed, with vertical reference lines at ±0.10 indicating the threshold for acceptable balance. CTO, chronic total occlusion; NYHA, New York Heart Association; PCI, Percutaneous coronary intervention; CABG, coronary artery bypass grafting; BMI, body mass index.
